# Prostaglandin E_2_ Prevents Hyperosmolar-Induced Human Mast Cell Activation through Prostanoid Receptors EP_2_ and EP_4_

**DOI:** 10.1371/journal.pone.0110870

**Published:** 2014-10-20

**Authors:** Ivonne Torres-Atencio, Erola Ainsua-Enrich, Fernando de Mora, César Picado, Margarita Martín

**Affiliations:** 1 Unidad de Farmacología, Facultad de Medicina, Universidad de Panamá, Panama, Panama Republic; 2 Laboratori d'Immunoal·lèrgia Respiratòria Clínica i Experimental, Institut d’Investigacions Biomèdiques August Pi i Sunyer (IDIBAPS), Barcelona, Spain; 3 Unitat de Bioquímica i Biologia Molecular, Department de Ciències Fisològiques I, Facultat de Medicina, Universitat de Barcelona, Barcelona, Spain; 4 Department de Farmacologia, Terapéutica i Toxicologia, Universitat Autònoma de Barcelona, Barcelona, Spain; 5 Centro de Investigaciones Biomédicas en Red de Enfermedades Respiratorias (CIBERES), Instituto de Salud Carlos III, Madrid, Spain; Universidade Federal do Rio de Janeiro, Brazil

## Abstract

**Background:**

Mast cells play a critical role in allergic and inflammatory diseases, including exercise-induced bronchoconstriction (EIB) in asthma. The mechanism underlying EIB is probably related to increased airway fluid osmolarity that activates mast cells to the release inflammatory mediators. These mediators then act on bronchial smooth muscle to cause bronchoconstriction. In parallel, protective substances such as prostaglandin E_2_ (PGE_2_) are probably also released and could explain the refractory period observed in patients with EIB.

**Objective:**

This study aimed to evaluate the protective effect of PGE_2_ on osmotically activated mast cells, as a model of exercise-induced bronchoconstriction.

**Methods:**

We used LAD2, HMC-1, CD34-positive, and human lung mast cell lines. Cells underwent a mannitol challenge, and the effects of PGE_2_ and prostanoid receptor (EP) antagonists for EP_1–4_ were assayed on the activated mast cells. Beta-hexosaminidase release, protein phosphorylation, and calcium mobilization were assessed.

**Results:**

Mannitol both induced mast cell degranulation and activated phosphatidyl inositide 3-kinase and mitogen-activated protein kinase (MAPK) pathways, thereby causing de novo eicosanoid and cytokine synthesis. The addition of PGE_2_ significantly reduced mannitol-induced degranulation through EP_2_ and EP_4_ receptors, as measured by beta-hexosaminidase release, and consequently calcium influx. Extracellular-signal-regulated kinase 1/2, c-Jun N-terminal kinase, and p38 phosphorylation were diminished when compared with mannitol activation alone.

**Conclusions:**

Our data show a protective role for the PGE_2_ receptors EP_2_ and EP_4_ following osmotic changes, through the reduction of human mast cell activity caused by calcium influx impairment and MAP kinase inhibition.

## Introduction

Asthma is a complex chronic inflammatory disease of the airways that involves the activation of many inflammatory and structural cells. Each component releases inflammatory mediators that result in the pathophysiological changes of typical of the condition [1]. Human mast cells (HuMC) are recognized as the key effector cells of allergic and non-allergic inflammation in asthma [2]. In addition to allergens, many non-immunological stimuli activate complex signaling cascades in mast cells that lead to the secretion of a plethora of autacoid mediators, cytokines, and proteases [3].

Exercise-induced bronchoconstriction (EIB) is a condition in which vigorous physical activity triggers acute airway narrowing. EIB occurs in response to a loss of water from the airways caused by hyperventilation associated with exercise. The osmotic theory proposes that the primary effect of airway water loss is the induction of an increased osmolality in the airway surface liquid [4] that stimulates the release of various mediators via mast cell mechanisms. Both the epithelium and eosinophils may be involved in the generation of EIB-related mediators [5], [6].

Experimental surrogates for exercise include the inhalation of hyperosmolar agents and mannitol drug powder [7]. The mannitol challenge is an indirect bronchial challenge [8], which exerts an osmotic effect on the airways and consequently has the potential to lead to mast cell activation [7], [9], [10], [11]. Thus, it can mimic the effects of exercise on airway fluid osmolarity.

Prostaglandin E_2_ (PGE_2_) is a product of the cyclooxygenase pathway of arachidonic acid metabolism that is produced in mast cells, dendritic cells, epithelial cells, fibroblasts, and macrophages. Clinical studies have shown that experimental treatment with PGE_2_ prevents allergen-, exercise-, and aspirin-induced airway obstruction [12], [13]. Furthermore, several studies have shown a link between asthmatic patients and low levels of PGE_2_ in isolated airway cells [14], [15], [16], suggesting a homeostatic role for PGE_2_ in the control of airway reactivity and/or inflammation.

PGE_2_ is a highly pluripotent prostanoid displaying a wide range of pro-inflammatory and anti-inflammatory effects in several tissues. Although PGE_2_ is a potent pro-inflammatory mediator [17], its role as an anti-inflammatory mediator is now being studied [18], [19]. In this context, it opposes the host inflammatory response, which potentially limits collateral damage to neighboring cells and tissues, thereby aiding the resolution of inflammation [20]. This dual effect appears to be dependent on the cell type, the tissue compartment, the state of cellular activation, and the expression pattern of four prostanoid (EP) receptor subtypes [21].

The EP receptors are members of the G protein-coupled receptor (GPCR) family. EP_1_ signals through Gαq, which increases Ca^2+^ levels. EP_2_ and EP_4_ signal through Gαs to increase cyclic-AMP (cAMP) levels, while EP_3_ primarily signals through Gαi to decrease cAMP levels. Further diversity among EP receptors is generated in both the EP_1_ and EP_3_ receptors by alternatively spliced C-terminal variants, as discussed elsewhere [22]. The EP_2_ receptor can downregulate antigen-mediated mast cell responses through Gαs-dependent production of cAMP, whereas the EP_3_ receptor can up-regulate antigen-mediated mast cell responses through enhanced calcium-dependent signaling [23], [24]. It has been suggested that differences in EP_2_ and EP_3_ receptor expression in mast cells could dictate the upregulation or downregulation of antigen-mediated responses by PGE_2_. Thus, the distribution and relative expression of these four receptor subtypes provide a flexible system describing the ability of PGE_2_ to evoke pleiotropic, sometimes opposing, tissue and cell actions [25]. Notably, the beneficial in vivo effects of PGE_2_ in murine models of allergic asthma might be mediated through EP_2_ receptors in airway mast cells [26], [27].

This study aimed to evaluate how PGE_2_ modulates the response to mannitol through prostanoid receptors as a model of exercise-induced asthma in human mast cells, and to clarify the related signaling events.

## Materials and Methods

### Cells, Antibodies, and Reagents

The LAD2 HuMC line, provided by Drs A Kirshenbaum and DD Metcalfe (National Institutes of Health, Bethesda, MD), was grown in StemPro-34 serum-free medium (Invitrogen Life Technologies, Carlsbad, California), supplemented with StemPro-34 nutrient, L-glutamine (2 mM), penicillin (100 U/ml), streptomycin (100 mg/ml), and 100 ng/ml recombinant stem cell factor (SCF) (Amgen, Thousand Oaks, California) as described elsewhere [28]. The human mast cell line 1 (HMC-1) was obtained from JH Butterfield (Mayo Clinic, Rochester, Minnesota), and was cultured in RPMI 1640 medium containing 10% (v/v) fetal bovine serum and 1% (v/v) penicillin/streptomycin at 37°C in 5% CO_2_ as decribed elsewhere [29].

Primary HuMCs derived from CD34-positive peripheral blood cells were obtained from National Institutes of Health by a Material Transfer Agreement #2009-0776. Healthy donors gave written informed consent. The National Institute of Allergy and Infectious Diseases Institutional Review Board and Ethics Committee approved the protocol (98-I-0027; principal investigator: Dr A Kirshenbaum). Primary HuMCs were differentiated in vitro for 8 weeks in the presence of 100 ng/ml interleukin (IL)-6 and 100 ng/ml SCF, as described previously [30]. We used the following antibodies: monoclonal anti-β-actin-peroxidase (Sigma-Aldrich, St. Louis, MO); anti-p38 Thr180/Tyr182, anti–p-ERK (i.e., extracellular-signal-regulated kinase 1/2) Thr202/Tyr204, and anti–p-JNK (i.e., c-Jun N-terminal kinase) Thr183/Tyr185 (Cell Signaling Technology, Danvers, MAs); pAKT antibody was from Santa Cruz Biotechnology, Santa Cruz, CA); PGE_2_ and antibodies against the EP (Prostaglandin E_2_) receptors were from Cayman Chemical (Ann Arbor, MI), except for EP-4 (Abcam, Cambridge, UK); EP_1_/EP_2_ antagonist receptor AH6809 and EP_4_ antagonist receptor AH23848 (Sigma); and EP_3_ antagonist receptor L-826266 (Merck, Darmstadt, Germany).

### Human Lung Mast Cells Purification and Culture

Mast cells were isolated from lung tissue obtained from patients undergoing lung resection for lung cancer. The study was approved by the Hospital Clinic Committee on Human Clinical Research, and by the Ethics Committee, (expedient number: 2012/7613, Hospital Clinic, Barcelona) and written informed consent was obtained from all patients. Using immunoaffinity magnetic selection, human lung mast cells (HLMCs) were dispersed from macroscopically normal lung obtained within 1 hour of resection from lung cancer patients, as described previously [31]. Final mast cell purity and viability were each 99%. HLMCs were cultured in Dulbecco's modified Eagles medium, 10% FCS, antibiotic/antimycotic solution, SCF (100 ng/ml), IL-6 (50 ng/ml), and IL-10 (10 ng/ml) [31].

### Beta-hexosaminidase release assay

HuMCs were stimulated with 10% mannitol in Tyrode’s buffer (10 mM HEPES, 137 mM NaCl, 2.7 mM KCl, 0.4 mM NaH_2_ PO_4_, 1.8 mM CaCl_2_, 1.3 mM MgSO_4_, 5.6 mM glucose, and 0.025% BSA) for 30 minutes at 37°C. For PGE_2_ stimulation and EP receptor antagonist assays we pre-incubated cells with a high dose of the antagonists for 10 minutes, before incubating with increasing PGE_2_ doses for 10 minutes each. Afterwards, cells were activated with mannitol 10% for 30 minutes. We used the following EP receptor antagonists: AH6809 (10 µM) blocking receptors EP_1_ and EP_2_ [32], L826266 (30 µM) blocking receptor EP_3_ [33], and AH23848 (10 µM) blocking receptor EP_4_ [34]. Mast cell degranulation was monitored by β-hexosaminidase release, as described previously [35]. The resulting β-hexosaminidase activity was expressed as the percentage of maximum response (samples treated with triton X-100), that is β-hexosaminidase release (%)  =  [(sample release − spontaneous release)/(maximum release − spontaneous release)]×100.

### Calcium mobilization

Calcium mobilization in LAD2 cells was followed by fluorimetric analysis of cytoplasmic-free calcium with Fluo-4 AM fluorescent dye (Molecular Probes, Invitrogen) as described elsewhere [36]. Briefly, 0.2×10^6^ cells/point were loaded with 5 mM Fluo-4-AM for 30 minutes at 37°C in the dark, washed twice with Tyrode’s buffer, and resuspended. To measure calcium influx in the absence of extracellular calcium, cells were washed and resuspended with Tyrode’s buffer without calcium. Fluorimetric measurements were by a Modulus II Microplate Multimode Reader (Turner Biosystems, Promega, CA), according to the manufacturer’s instructions. After defining basal conditions the stimuli was added (time 0) and fluorimetric measures were done 10 more minutes. Each point was done by triplicate.

### ELISA for PGE_2_

2×106 cells were activated with 10% mannitol for 30 minutes and 4 hours. Supernatants were collected and concentrations of PGE_2_ were measured with enzyme immunoassay kits (Cayman Chemical, Ann Arbor, Mich) according to the manufacturer’s instructions.

### Cell activation and immunoblotting

Cells were treated in Tyrode’s buffer for 15 minutes with either 10% mannitol stimulation and/or PGE_2_ at 10 µM. EP receptor antagonist pretreatment was done 10 minutes before any cell stimulation as follows: AH6809 (10 µM) blocking receptors EP_1_ and EP_2_ [32], L826266 (30 µM) blocking receptor EP_3_ [33], and AH23848 (10 µM) blocking receptor EP_4_ [34]. For immunoblotting experiments, cells were washed twice with ice-cold PBS and dissolved in a lysis buffer (1% Triton X-100, 50 mM Tris [pH 7.4], 150 mM NaCl, 20 mM octyl-b-glucoside, 100 mM NaF, 1 mM Na_3_VO_4_, 1 mM PMSF 1 mM sodium pyrophosphate, and protease inhibitor mixture; Roche Molecular Biochemical, Indianapolis, Indiana). Cell lysates were clarified by centrifugation. The total cell lysates were separated by sodium dodecyl sulfate polyacrylamide gel electrophoresis (SDS-PAGE) and electrotransferred to polyvinylidene difluoride membranes (Millipore, Bedford, Massachusetts). Blots were probed with the indicated antibodies, and proteins were visualized by enhanced chemiluminescence (Santa Cruz Biotechnology).

### RNA extraction and Real-time polymerase chain reaction

Total RNA was extracted with an RNAeasy Mini Kit (Qiagen, Hilden, Germany) from 2×10^6^ LAD2 cells. We generated complementary DNA from an mRNA High-Capacity cDNA Reverse Transcription Kit (Applied Biosystems) according to the manufacturer’s instructions. To amplify EP receptors from LAD2, the primer pairs detailed in Table 1 were used.

**Table 1:** Primers used to determine prostaglandin E_2_ receptor expression on LAD2 cells by quantitative PCR.

Gene Name	5′-3′ primer sequence	Accession Number
βactin	FW 5′GAAACTACCTTCAACTCCATC-3′ RW 5′-CTAGAAGCATTTGCGGTGGAC-3′	NM_001101
EP_1_ receptor	FW 5′-TGTCGGTATCATGGTGGTG-3′ RW 5′GGCCTCTGGTTGTGCTTAGA-3′	NM_000955
EP_2_ receptor	FW 5′-GTCTGCTCCTTGCCTTTCAC-3′RW 5′-CCTCAAAGGTCAGCCTGTTT-3	NM_000956
EP_3_ receptor	FW 5′-CCACCTTTTCTTCGCCTCTG-3′ RW 5′-TTTCTGCTTCTCCGTGTGTG-3′	NM_198715*
EP_4_ receptor	FW 5′-TCCAGCACCATTCTTCACTG-3′ RW 5′-AACAAAGTGCCCAACAGGTC-3′	NM_000958

*variant number 5 of the ten existing variants of the receptor.

### Real-time polymerase chain reaction

Real-time polymerase chain reaction (RT-PCR) for EP_1_, EP_2_, EP_3_, EP_4_, IL-8, and tumor necrosis factor (TNF) was performed using the TaqMan Gene Expression Assay (Applied Biosystems) on an ABI-Prism 7300 Sequence Detector (Applied Biosystems). 18S RNA amplification control was used for cycle normalization. Data were analyzed using the 7500 SDS Software (Applied Biosystems). All PCR reactions were set up in triplicate.

### Fluorescence-activated cell sorting

Flow Cytometry was by fluorescence-activated cell sorting (FACS). CD63 expression was detected by direct staining with fluorescein isothiocyanate anti-CD63 (Beckton Dickinson, Franklin Lakes, NJ) for 30 min at 4°C. Cells were then analyzed using a FACSCalibur flow cytometer (FACScan; BD Biosciences, Mountain View, CA).

### Statistical data analysis

All results are expressed as mean ± standard deviation (SD). After confirming the normality of the sample distribution and performing variance analysis, we used the Student *t* test to determine significant differences (*p* value) between two experimental groups.

## Results

### Mannitol-induced mast cell degranulation is a calcium dependent process

**Figure 1:**
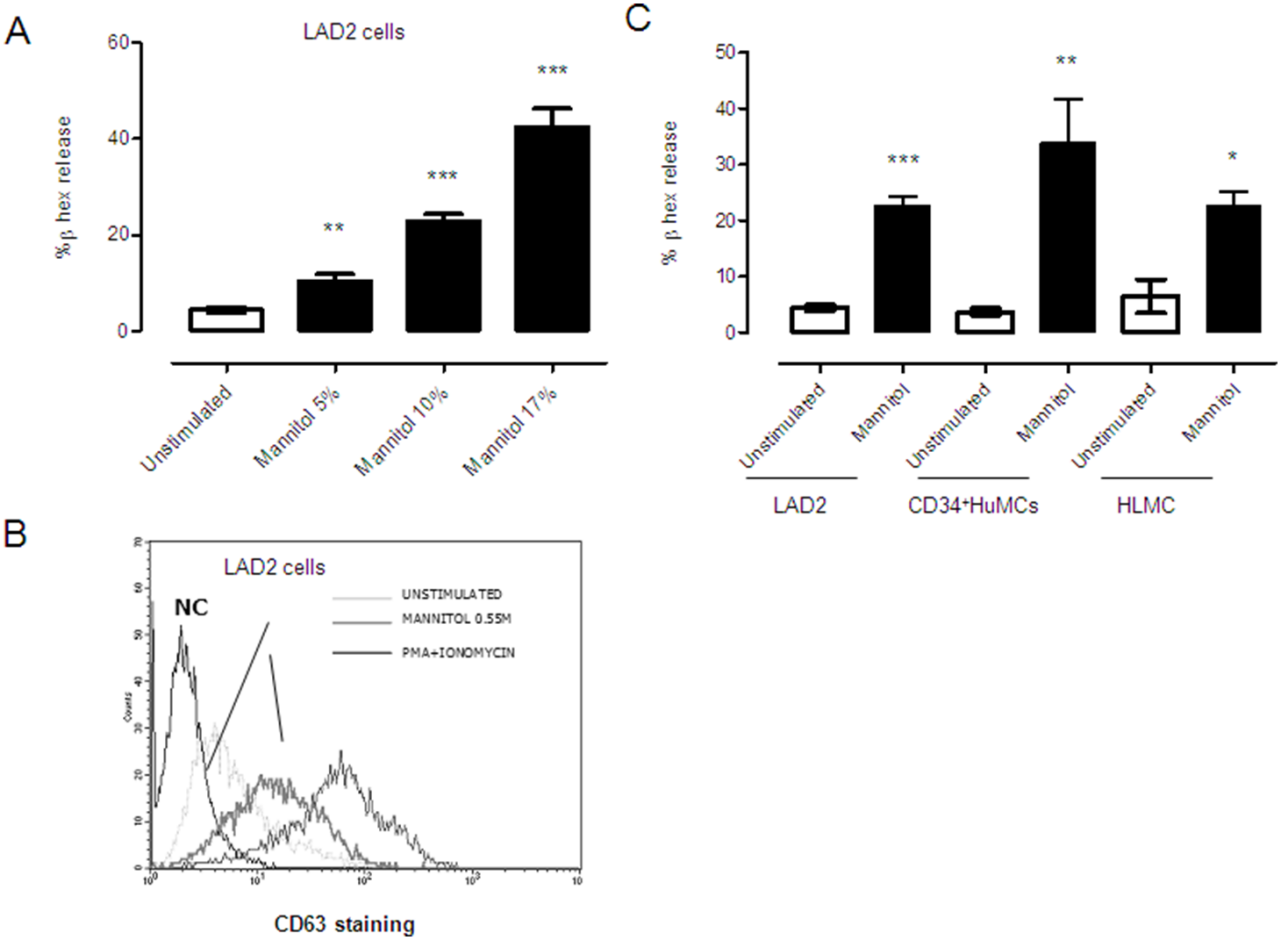
Mast cell degranulation is induced by mannitol in human mast cells. LAD2 mast cells were stimulated with mannitol (5%, 10%, and 17%) for 30 minutes, and β-hexosaminidase release was measured (A). CD63 staining was performed in LAD2 cells after mannitol stimulation (10%) for 30 minutes. Positive control was conducted with PMA plus ionomycin. Negative control (NC) means isotype staining (B). β-hexosaminidase content was measured in the supernatants from activated LAD2 cells, CD34+ derived human mast cells, and human lung mast cells induced by mannitol (10%) at 37°C for 30 minutes (C). Results are in triplicate and are the mean of three independent experiments expressed as mean ± standard deviation. Statistical significance *p≤0.05, **p≤0.01, ***p≤0.001) is relative to the unstimulated control cells.

Mannitol was chosen as a hyperosmolar agent because of its ability to induce mast cell degranulation. To obtain the optimal concentration of mannitol for mast cell activation, LAD2 cells were incubated with a range of concentrations for 30 minutes (Figure 1A). As shown, all mannitol concentrations caused degranulation; therefore, the intermediate concentration (10%) was chosen for further experiments. After mannitol treatment, the expressions of both β-hexosaminidase release and CD63 were measured by colorimetric assay and FACS, respectively, as markers of degranulation. FACS staining allowed us to distinguish degranulated cells and to discard dead cells with mannitol-induced toxic effects. Mannitol treated cells induced CD63 expression on the mast cell surface membrane (Figure 1B).

**Figure 2:**
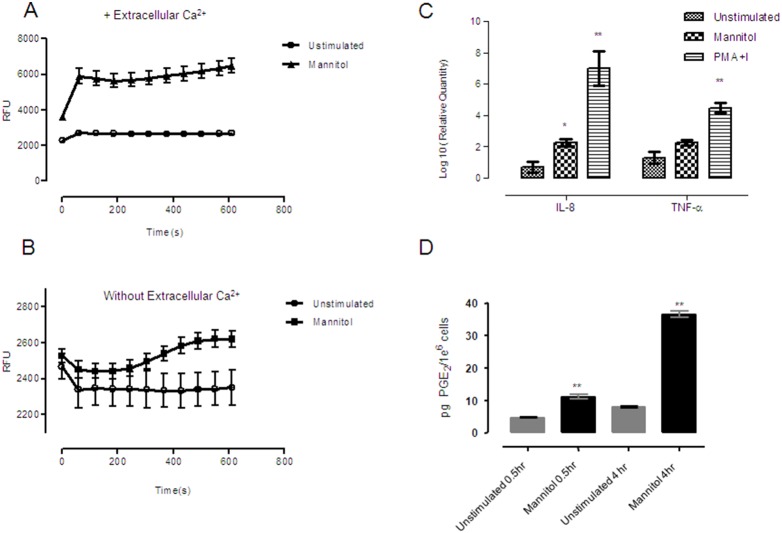
Calcium mobilization, cytokine production and PGE_2_ secretion are induced by mannitol in LAD2 cells. Determination of calcium flux following mannitol stimulation (10%) was performed in LAD2 cells loaded with Fluo-4 dye, either with calcium containing media (A) or without extracellular calcium (B), as described in material and methods section. After defining basal conditions the stimuli was added (time 0) and measured 10 minutes. Real time polymerase chain reaction was performed in LAD2 cells that were either unstimulated or stimulated with 10% mannitol, using interleukin-8 and tumor necrosis factor alfa as probes. PMA+ Ionomycin (Sigma) were used as positive control stimuli (C). PGE_2_ release was measured at different times as indicated in the figure using an ELISA assay. Results are expressed in pg per 1×10^6^ cells (D). Results are triplicates expressed as mean ± standard deviation, and are representative of three independent experiments. Statistical significance (* p≤0.05, **p≤0.01) is relative to unstimulated control cells. RFU: relative fluorescence units.

Next, we extended the study to CD34+ derived HuMC and HLMC and obtained similar results (Figure 1C). It has been described that degranulation induced by aggregation of high-affinity Immunoglobulin E receptor (FcεRI) is dependent on the influx of extracellular calcium across the cell membrane. In contrast, non-immunological secretagogues can induce degranulation independently of extracellular calcium [37]. Using fluorimetric analysis, our data show that mannitol was able to release calcium from both the extracellular (Figure 2A) and the intracellular compartments (Figure 2B). We next analyzed late mast cell responses by studying TNF and IL-8 production by RT-PCR after mannitol stimulation compared to PMA and ionomycin stimulation for 6 hours. We demonstrated that mannitol could trigger IL-8 and TNF production (Figure 2C). Interestingly, mannitol was able to induce PGE_2_ secretion as short as 30 minutes as we show in figure 2D. Collectively, the findings demonstrate that mannitol induced early and late events in mast cell activation.

### PGE_2_ down regulates mannitol-induced-degranulation in mast cells

**Figure 3:**
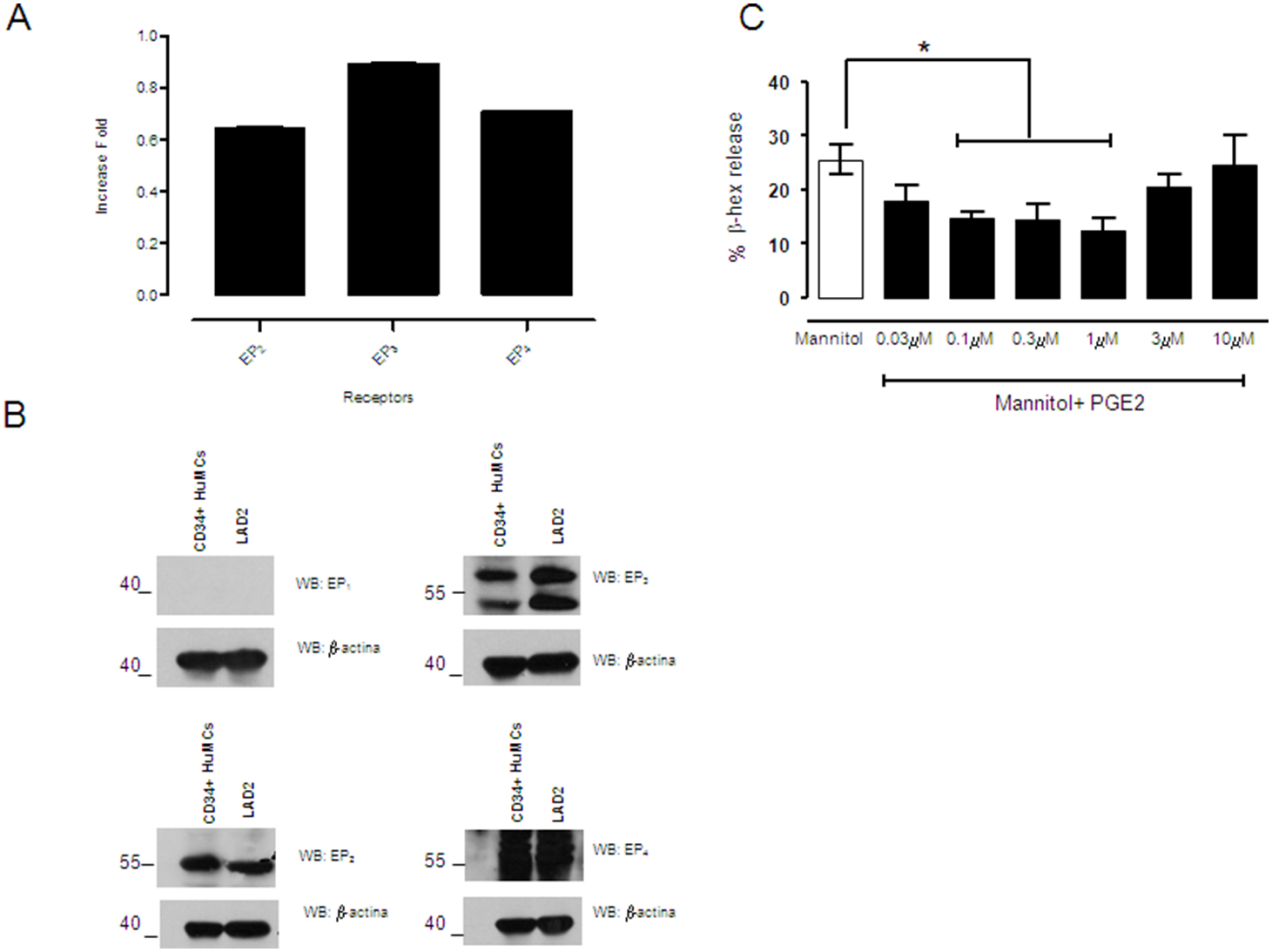
EP_2_, EP_3_, and EP_4_ are expressed on human mast cells. At low doses prostaglandin E_2_ inhibits mannitol mast cell degranulation. Real time PCR was performed in LAD2 cells using EP receptor probes as indicated in Table 1 (A). The EP receptors expression levels were normalized with the β-actin expression level, EP_1_ expression was undetectable. Western blot analysis was carried out with specific antibodies against EP_1_, EP_2_, EP_3_, and EP_4_ in whole cell lysates from CD34^+^ derived mast cells and LAD2 cells; blot against β-actin was performed as a loading control (B). PGE_2_ titration was carried out before 10% mannitol stimulation in LAD2 cells (C). The experiments are representative of 3 independent assays. Statistical significance (*p≤0.05) is relative to mannitol-stimulated cells.

Next, we studied the modulating effects of PGE_2_ on mannitol-induced degranulation in human mast cells. First, we performed quantitative PCR to determine the pattern of expression of EP receptors by LAD2 cells, and then assayed the LAD2 cell lysates by western blot using specific antibodies against the EP receptors. Our data indicate that LAD2 cells express EP_2_, EP_3_, and EP_4_ receptors, but not EP_1_ receptors (Figure 3A–B). Once the EP receptor pattern was established, we evaluated the effects of PGE_2_. EP receptor stimulation by PGE_2_ has been reported to enhance FcεRI-mediated mast cell degranulation via EP_3_ in micromolar ranges [38], although the positive effect of PGE_2_ seems to vary depending on the mast cell type [39]. Since we used an osmotic stimulus rather than an immunologic stimulus, both the mechanism and intensity may have differed. Therefore, we pre-incubated LAD2 cells with increasing doses of PGE_2_ (0.1, 1, and 10 µM) for 10 minutes each, before activating them with 10% mannitol for 30 minutes. A β-hexosaminidase assay was conducted showing that PGE_2_ significantly decreased mannitol-induced degranulation at lower doses (Figure 3C).

### PGE_2_ exerts a protective effect through EP_2_ and EP_4_ receptors after mannitol activation

**Figure 4:**
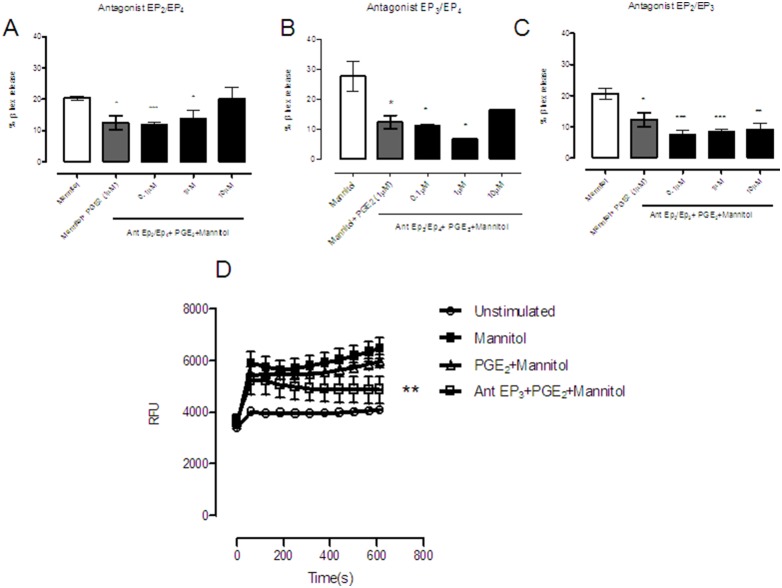
Mannitol-induced β-hexosaminidase release and calcium mobilization were altered in the presence of prostaglandin E_2_ and prostanoid receptor antagonists in LAD2 cells. β-Hexosaminidase release was conducted in mannitol-stimulated LAD2 cells pre-treated with antagonist to PGE_2_ receptors for 10 minutes and PGE_2_ at various doses (0, 1, 1, 10 µM). EP_2_ and EP_4_ receptor antagonists (AH6809, AH23848 at 10 µM), (A); EP_3_ and EP_4_ receptor antagonists (L826266 30 µM and AH23848 at 10 µM) (B); and, EP_2_ and EP_3_ receptor antagonists (AH6809 at 10 mM and L826266 at 30 µM) (C). Results are in triplicate and are the mean of three independent experiments expressed as mean ± standard deviation. Calcium mobilization was performed in cells that were left untreated or treated with 10% mannitol, 10% mannitol plus prostaglandin E_2_ (1 µM), or pretreated with an EP_3_ antagonist (L826266 at 30 µM) for 10 minutes and then stimulated with 10% mannitol and prostaglandin E (1 µM),_2_ (D). After defining basal conditions the stimuli was added (time 0) and measured 10 minutes. Statistical significance (*p≤0.05, **p≤0.01, ***p≤0.001) is relative to mannitol-stimulated cells.

To identify the EP receptors involved in the protective effect, antagonists of prostanoid receptors were assayed. Our results reveal that β-hexosaminidase release was only slightly decreased when low doses of PGE_2_ were used, but not at the highest concentration in EP_2_ and EP_4_ receptor antagonists pretreated cells (Figure 4A). Thus, it is unlikely that the EP_3_ receptor is responsible for the PGE_2_-induced reduction of mannitol-induced degranulation. Conversely, when the EP_2_ (Figure 4B) and EP_4_ (Figure 4C) receptors were free, and the EP_3_ receptors were antagonized, mediator release was significantly decreased regardless of concentration. In parallel, the EP_2_ and EP_4_ receptors mediated increases in cAMP through activation of adenylyl cyclase, while EP_3_ receptor has been shown to both inhibit and activate adenylyl cyclase as well as to drive calcium mobilization [21]. Consistent with these data, mannitol-induced calcium release was impaired when EP_3_ receptors were antagonized (Figure 4D). The data support a role for EP_3_ receptors in calcium influx triggered by PGE_2_ in mast cells.

**Figure 5:**
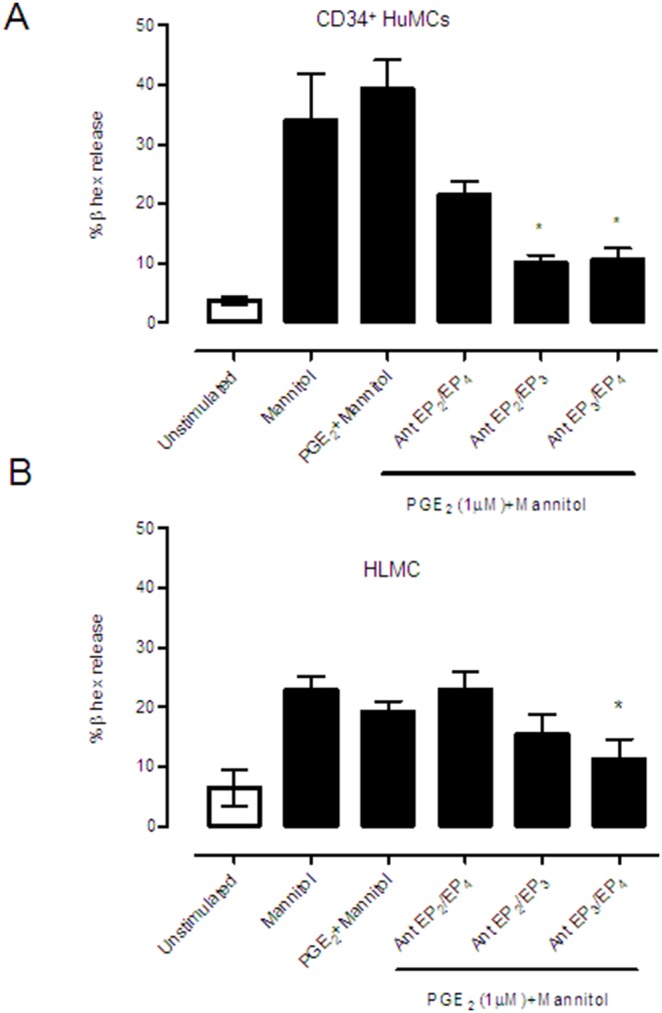
Prostaglandin E_2_ impairs mannitol-induced degranulation in human mast cells through the prostanoid receptors EP_2_ and/or EP_4_. Human CD34^+^ derived-mast cell (A) and human lung mast cell (B) degranulation was induced by 10% mannitol and prostaglandin E_2_ at 1 µM in the presence or absence of prostanoid receptor antagonists as indicated in the figure at doses indicated in material and methods section. Results are in triplicate and are the mean of three independent experiments expressed as mean ± SD. Statistical significance (*p≤0.05) is relative to mannitol-stimulated cells.

We extended these studies by examining the role of PGE_2_ after mannitol treatment in CD34+ derived mast cells and HLMC. As shown in Figure 5, HuMC and LAD2 cell lines responded similarly, suggesting significant protection when PGE_2_ acted via the EP_2_ and EP_4_ receptors in CD34+ derived-mast cells (Figure 5A), and that the EP_2_ receptor appears responsible for PGE_2_-driven protection in HLMC (Figure 5B).

### PGE_2_ interferes with Phosphatidyl Inositide 3-Kinase and Mitogen-Activated Protein Kinase Signaling after Mannitol Stimulation

**Figure 6:**
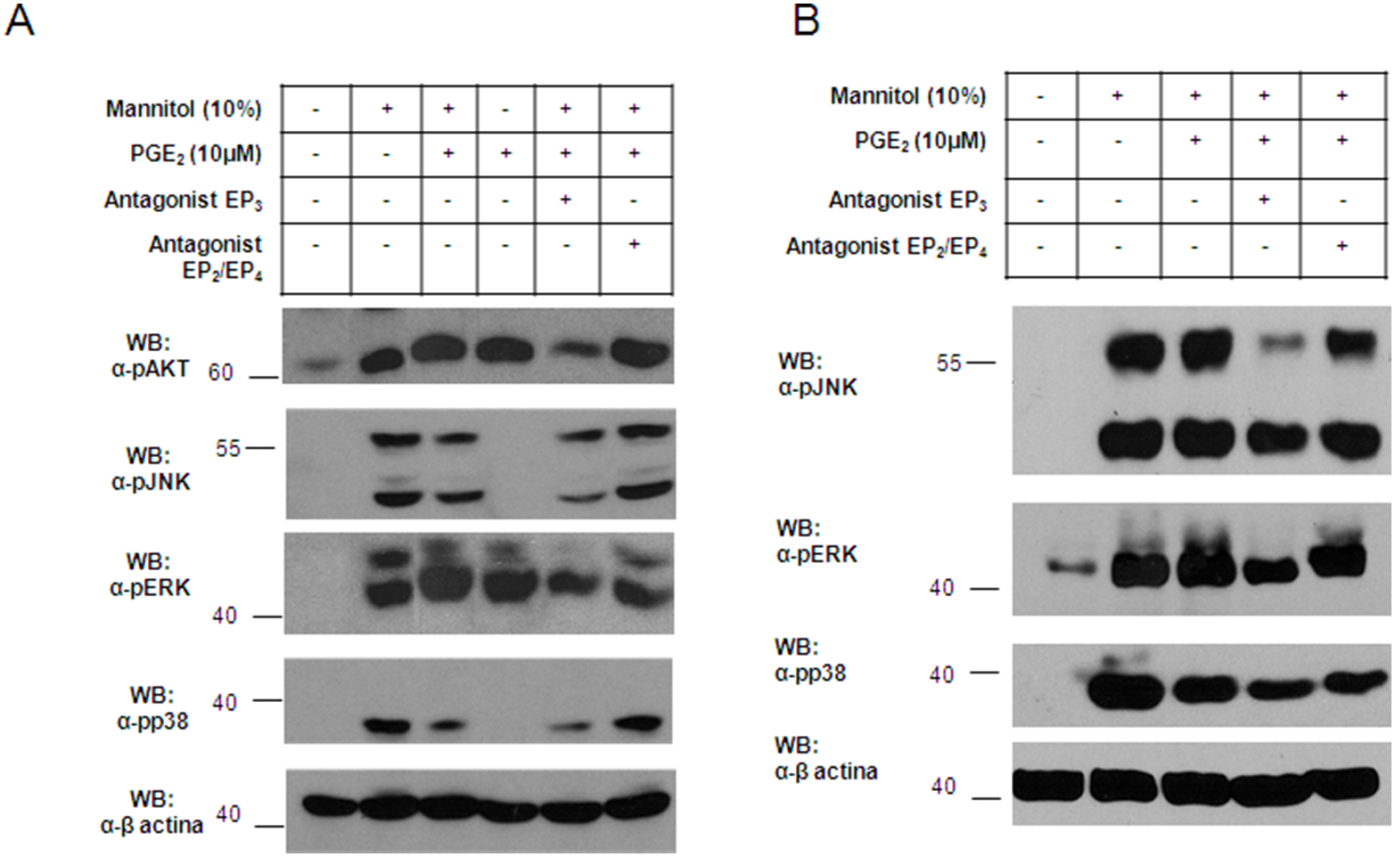
Phosphatidyl inositide 3-kinase (PI3K) and mitogen-activated protein kinase (MAPK) pathways are modulated by prostaglandin E_2_ in mannitol-stimulated mast cells. Cells were treated in Tyrode’s buffer for 15 minutes with either 10% mannitol stimulation and/or PGE_2_ at 10 µM. EP receptor antagonist pretreatment was done 10 minutes before any cell stimulation as follows: AH6809 (10 µM) blocking receptors EP_1_ and EP_2_, L826266 (30 µM) blocking receptor EP_3_, and AH23848 (10 µM) blocking receptor EP_4_. Western Blot analysis was performed in LAD2 cells (A) and HMC-1 (B) to explore (1) AKT phosphorylation as a surrogate marker for PI3K activation, and (2) MAPK activation via JNK, ERK1/2, and p38 phosphorylation. Blot against β-actin was performed (as a loading control). The data is representative of three independent experiments.

We assessed the effects of PGE_2_ in the phosphorylation of proteins after mast cell activation by osmotic changes. To do so, we examined the phosphatidyl inositide 3-kinase (PI3K) and mitogen-activated protein kinase (MAPK) pathways by western blot analysis in LAD2 (Figure 6A) and HMC-1 cells (Figure 6B). The phosphorylation status of AKT (Protein Kinase B) was assayed as a surrogate marker for PI3K activation. AKT phosphorylation was decreased under conditions where the EP_3_ receptor was blocked in mannitol treated cells, indicating a decrease in PI3K activation/OK (Figure 6A). AKT was constitutively increased in HMC-1 cell lines in which the KIT receptor was mutated and delivered signals independently of ligand engagement (data not shown). PGE_2_ prevented mannitol-induced phosphorylation of ERK1/2, p38, and JNK in LAD2 and HMC-1 cell lines when the EP_3_ receptor was blocked (Figure 6A, B). Together, these results indicate that PGE_2_ exhibits inhibitory effects on mannitol-induced osmotic activation by binding to EP_2_ or EP_4_ receptors in human mast cells.

## Discussion

This study aimed to evaluate the protective effect of PGE_2_ on mannitol-induced mast cell activation as a model of EIB in Asthma, where mannitol was used as a hyperosmolar stimulus. The use of a hypertonic agent stems from the theory that EIB is caused by increased osmolarity of the surface of the airways through the release of proinflammatory mediators [8]. Previous in vitro work on HLMCs showed that hyperosmolar stimulation induced histamine release, suggesting that hyperosmolar mediated release was a mechanism by which exercise-induced hyperventilation might induce asthma [40]. Our results show that mannitol induces mast cell signaling events that are possibly involved in the inflammatory response observed in asthma.

At early stages, mannitol increased degranulation in a calcium dependent manner before IL-8 and TNF alfa production occurred. Mannitol triggered the activation of PI3K and MAPK cascades, which enhanced ERK1/2, p38 and JNK phosphorylation. The MAPK pathway activates transcription factors such as AP-1 that in turn regulate cytokine and metalloprotease production [41]. Additionally ERK1/2 phosphorylates cytoplasmic phospholipase A2, which is involved in the production of the eicosanoid precursor arachidonic acid [42], [43]. Interestingly, previous studies reported ERK phosphorylation in airway smooth muscle cells that cause increased production of both IL-1β and granulocyte-macrophage colony-stimulating factor, which are involved in the contractile response and remodeling of the airways in asthma [43]. The role of JNK in asthma is related to extracellular matrix deposition, with its activation causing the release of growth factors such as transforming growth factor beta, which may explain the phenotype transition from fibroblasts to myofibroblasts in the lung [44]. Moreover, p38 regulates the antigen-triggered migration of mast cells and mediates the production of IL-8.

PGE_2_ is a highly pluripotent prostanoid displaying a wide range of effects, including smooth muscle relaxation and contraction, and both pro-inflammatory and anti-inflammatory properties [21]. These opposing effects are possible due to the presence of at least four subclasses of EP receptors (EP_1–4_) [45]. It has been reported that CD34+ derived mast cells express the PGE_2_ receptors EP_2_, EP_3,_ and EP_4_ [38]. Our data shows that the LAD2 cell line has a similar PGE_2_ receptor pattern.

The aim of the study was to evaluate how PGE_2_ modulates the response to mannitol through prostanoid receptors as a model of exercise-induced asthma in human mast cells. For that reason we used antagonist of the receptor instead of direct agonist of them. It has to be noted that AH6809, antagonist for EP_2,_ is also known to interact with DP1, and AH23848, a EP_4_ antagonist, can interact also with the TP receptors. DP1 has been suggested to be expressed on murine mast cells having a role on murine mast cell maturation and differentiation [46]. In our experiments, we are dealing basically with mature human mast cell systems subject to a short term incubation with AH6809. No such maturation effect is expected under our circumstances/conditions. Regarding AH23848, there is very little information on the presence of TP receptors on the human mast cells surface. In fact, it has been reported that the TP agonits U-46619 has no effect on human mast cells [47].

We found that when PGE_2_ triggers the EP_3_ receptor, it exerts a limited protective effect on mannitol-induced mast cell degranulation. In contrast, when PGE_2_ acts through EP_2_ and EP_4_ receptors, mannitol-induced mast cell degranulation and calcium influx are significantly nullified. Our data agree with other studies in which PGE_2_ has been shown to work through EP_2_ receptors to stabilize lung mast cells after IgE dependent activation [21], [47] and with studies reporting that the EP_2_ agonist butaprost exerts a protective effect in allergen-sensitized mice [27]. Additionally, a recent study using human bronchial smooth muscle proposes that PGE_2_-induced relaxation is mediated via the EP_4_ receptor [48], which contrasts with reported role of the EP_3_ receptor in the induction of PGE_2_ airway irritability and cough [49].

Gαs, the EP_2_ and EP_4_ receptor stimulation protein, results in adenylate cyclase activation and intracellular cAMP production. Conversely, EP_3_ receptor signaling is predominantly coupled to protein Gαi and produces reduced cAMP levels [50]. The accumulation of cAMP promoted by EP_2_ and EP_4_ receptors is associated with inhibition of cell function, whereas intracellular calcium increases induced by the EP_3_ receptor are linked to cellular activation [51]. The evidence from this study, along with other reports, supports the notion that PGE_2_ stabilizes mast cells through the EP_2_ and/or EP_4_ receptors, thereby providing control of the deleterious effects of mast cell degranulation in the airways. The presence of various EP_3_ isoforms could explain the differential release of mediators in degranulation assays at different PGE_2_ concentrations. It has been reported that, by interacting with the EP_3_ receptor, higher doses of PGE_2_ increase mediator release through IgE dependent mechanisms [52]. In addition, the presence of several EP_3_ isoforms might explain the protective effects of EP_3_ in suppressing allergic inflammation in mice [53]. Additionaly, it should be noted that the EP receptors expression pattern has been reported to be different in murine mast cells. EP_1_, EP_3_, and EP_4_ transcripts have been found in IL-3-dependent murine mast cell line, MC/9 [54] and murine bone marrow derived mast cells [55]. but not EP_2_. Our data in LAD2 cells is supported by the data obtained in CD34+ derived cells and HLMCs where the decrease in mannitol-induced degranulation was significant when the EP_2_ and EP_4_ receptors were free to interact with PGE_2_.

The mannitol stimulus caused increased activation in both MAPK and PI3K signaling pathways in mast cells. PGE_2_ modulated the mannitol phosphorylation profile of these pathways differently according to the receptor that was triggered. Thus, when the EP_3_ receptor was involved, ERK1/2, p38, and JNK phosphorylation remained active, while their phosphorylation decreased with EP_2_ or EP_4_ receptor engagement. Our results suggest that PGE_2_ is not only able to modulate early mast cell events through degranulation, but that it can regulate downstream events that may perpetuate airway inflammation in diseases such as asthma.

Experimental treatment with PGE_2_ prevents exercise–induced airway obstruction [13]. The preventive effects of exogenous effects of PGE_2_ on EIB might suggest that an insufficient biosynthesis of endogenous PGE_2_ during exercise in asthma patients can contribute to exercise-induced bronchoconstriction. Interestingly, exercise increases PGE_2_ release in the airways of healthy subjects [56], but this increase is not detected in asthma patients [57].

In conclusion, we have provided functional in vitro evidence that EP_2_/EP_4_ are potential therapeutical targets having a role in the regulation of MC degranulation that may prevent EIB in asthma by nullifying the hyperosmolar-induced degranulation of airway mast cells.
